# First Report of* Chlamydia abortus* in Farmed Fur Animals

**DOI:** 10.1155/2018/4289648

**Published:** 2018-11-26

**Authors:** Zhaocai Li, Ping Liu, Xiaoan Cao, Zhongzi Lou, Kinga Zaręba-Marchewka, Monika Szymańska-Czerwińska, Krzysztof Niemczuk, Bo Hu, Xue Bai, Jizhang Zhou

**Affiliations:** ^1^State Key Laboratory of Veterinary Etiological Biology, Lanzhou Veterinary Research Institute, Chinese Academy of Agricultural Sciences, Lanzhou, China; ^2^Center for Biomedical Research, Northwest Minzu University, Lanzhou, China; ^3^Department of Cattle and Sheep Diseases, National Veterinary Research Institute, Pulawy, Poland; ^4^Laboratory of Serological Diagnosis, National Veterinary Research Institute, Pulawy, Poland; ^5^Institute of Special Animal and Plant Sciences, Chinese Academy of Agricultural Sciences, Changchun, China

## Abstract

*Chlamydia (C.) abortus*, a globally distributed obligate intracellular bacterium, has attracted increasing interest according to its veterinary importance and zoonotic nature.* C. abortus* can infect a variety of animals and cause foetal loss in livestock resulting in economic loss. In this study, the samples collected from two farms of foxes (n=20), raccoon dogs (n=15) and minks (n=20), were investigated by* Chlamydiaceae*- and* Chlamydia* species-specific real-time PCR. The results showed that all the tested foxes (20/20) and raccoon dogs (15/15) harbored* Chlamydia* spp., while 5% of minks (1/20) were positive for* Chlamydia* spp.* C. abortus* was identified in all positive samples as the dominant* Chlamydia* species, with* C. pecorum* DNA coexistence in some of the rectal samples (7/20) taken from foxes. Phylogenetic analysis based on specific gene fragments of 16S rRNA, IGS-23S rRNA, and* ompA* revealed that all sequences obtained in this study were assigned to the* Chlamydiaceae* family with high similarity to* C. abortus* S26/3 and B577 previously identified in ruminants. This is the first report confirming that farmed foxes, raccoon dogs, and minks carry* C. abortus*. Further studies are needed to fully elucidate the epidemiology and pathogenicity of this pathogen in farmed fur animals as well as the potential risks to public health.

## 1. Introduction

*Chlamydia* spp. are obligate intracellular bacteria widely distributed throughout the world and responsible for a variety of diseases in humans and animals. To date, eleven species have been identified along with five newly characterized candidate species in the single genus of the family* Chlamydiaceae* [1–5].* Chlamydia abortus *(*C. abortus*) has attracted increasing interest due to its veterinary importance and zoonotic potential.* C. abortus* can infect a variety of mammalian hosts and is associated with abortion cases in ruminants, pigs, horses, rabbits, guinea pigs, and mice [6]. In addition, infection with* C. abortus* in nonmammalian hosts has also been reported recently [7, 8]. Infections of* C. abortus* are common in ruminants such as sheep, goats, and cattle in many countries around the world, causing tremendous economic loss for the livestock industry [9]. Infected animals may be a source of infection in humans and lead to severe outcomes including respiratory disorder and miscarriage [10, 11]. The wide distribution, broad-host infection, and zoonotic potential of* C. abortus* may exhibit an increasing but neglected risk to public health. Therefore, there is an urgent need to investigate the presence of this zoonotic pathogen in animals closely related to humans and evaluate the potential health risk.

Foxes, raccoon dogs, and minks are captive-bred wild animals raised as fur animals with substantial populations in northern Europe, northern America, and China [12]. These animals are suspected to be infected with a wide range of pathogens including several important zoonotic ones, such as rabies virus, hepatitis E virus, and influenza virus [13–15]. However, there are as yet no reports on the prevalence of chlamydiosis in these animals. Indeed, reproductive failure cases of abortion and stillbirth in foxes, raccoon dogs, and minks were commonly observed in some farms with unknown reasons in Northeast China. Thus, a survey was conducted in China to acquire information concerning farmed fur animal species as possible reservoirs of* Chlamydia* spp.

## 2. Materials and Methods

Samples from fur animals came from two farms located in the suburbs of Changchun city, Jilin province, China, and sampling was performed in November 2017 in a local slaughterhouse. All samples were collected before the slaughtering of the animals and immediately transported on ice to the laboratory. A formal ethical approval is not required for this kind of study and sampling was performed by authorized veterinarians during routine medical and veterinary activities. A total of 55 sampled animals included 9 male and 11 female foxes, 12 male and 3 female raccoon dogs, and 16 male and 4 female minks (Table 1). In total, rectal swabs (n=55), whole blood (n=55) samples, conjunctive swabs (n=35), and vaginal swabs (n=18) were collected from apparently healthy minks, while the foxes and raccoon dogs came from farm with an abortion history. Samples were stored at −80°C until use. DNA extraction from blood (200 *μ*l), vaginal swabs, and conjunctive swabs was performed using the commercial TIANamp Genomic DNA Kit (Tiangen Biotech Co., LTD) following the manufacturer's protocol. For rectal swabs, the QIAamp DNA Stool Mini Kit (Qiagen) was used for DNA extraction. All DNA samples were eluted in 100 *μ*l of elution buffer and screened using a* Chlamydiaceae*-specific real-time PCR targeting the 23S rRNA gene fragment, as previously described by Ehricht* et al.* [16]. All* Chlamydiaceae*-positive samples were retested with specific real-time PCR assays to identify* Chlamydia* species including* C. abortus*,* C. pecorum*,* C. psittaci*,* C. felis*, and* C. suis* [17]. An analytical cut-off value of 39.0 was selected corresponding to the defined lower limit of detection of the test. Any Ct value above this defined limit would, thereafter, be considered unreliable. For further quantification DNA concentration from the positive samples, the target fragments for each detecting primer pairs were chemically synthesized and cloned into pMD18T vector (Takara). Quantification of the recombinant plasmids was done on a Nanodrop ND-2000 (Thermo, US), and 10-fold dilutions (6×10^7^ copies to 6×10^1^ copy/*μ*l) were used as positive controls to establish a standard curve for quantification. The* Chlamydia* DNA copies in the samples were tested and calculated according to the formulation obtained from the standard curves.* Chlamydiaceae* positive DNA samples with a high copies based on real-time PCR were used for DNA sequencing to confirm the identity of* Chlamydia *spp. Specific fragments of* ompA*, 16S rRNA, and 16S rRNA-23S rRNA intergenic spacer together with 23S rRNA domain I (IGS-23S rRNA) were amplified with previously published primer sets [8]. PCR amplicons were sent to an external company (Shanghai Sangon Biotech, China) for sequencing. The obtained sequences were deposited in the GenBank database with the following accession numbers: MH532474-MH532478 (16S rRNA), MH537631-MH537633 (IGS-23S rRNA), and MH542157-MH542161 (*ompA*).

The data were analysed using MEGA 5.05 software [18]. Amplicons were subjected to BLAST analysis against the GenBank database (NCBI) to identify related entries and aligned with a panel of* Chlamydia *reference strains. To assess the phylogenetic relationship between* Chlamydia* spp. and the tested samples, phylogenetic trees for 16S rRNA (1358bp) and IGS-23S rRNA (990 bp) as well as* ompA* (940 bp) were constructed by the neighbor-joining method with 1,000 replicates' bootstrap using the Maximum Composite Likelihood model with MEGA 5.05.

## 3. Results and Discussion

The results of real-time PCR detection are presented in Table 1 and Table S1. Among 55 tested animals, 36 were shedders of* Chlamydiaceae* giving a prevalence rate of 65.45%. Foxes and raccoon dogs demonstrated the highest level of* Chlamydiaceae* prevalence at 100%. Positive results for* Chlamydiaceae* were obtained for all rectal swabs from foxes and raccoon dogs irrespective of gender. DNA of* Chlamydiaceae* was also present in several blood (n=10) and conjunctive swab (n=24) samples from foxes and raccoon dogs. For the females, vaginal swab samples revealed a high rate of positive results for* Chlamydiaceae* in foxes (9/11) and raccoon dogs (1/3). Further testing with species-specific real-time PCR identified the detected agent in all* Chlamydiaceae* positive samples as* C. abortus*. Moreover in 7 rectal swabs from foxes the coexistence of* C. pecorum* was confirmed. No other* Chlamydia* species was identified. Only one rectal swab from a male mink was tested positive for* Chlamydiaceae* and determined as* C. abortus* with a relatively low level of DNA copies.

**Figure 1:**
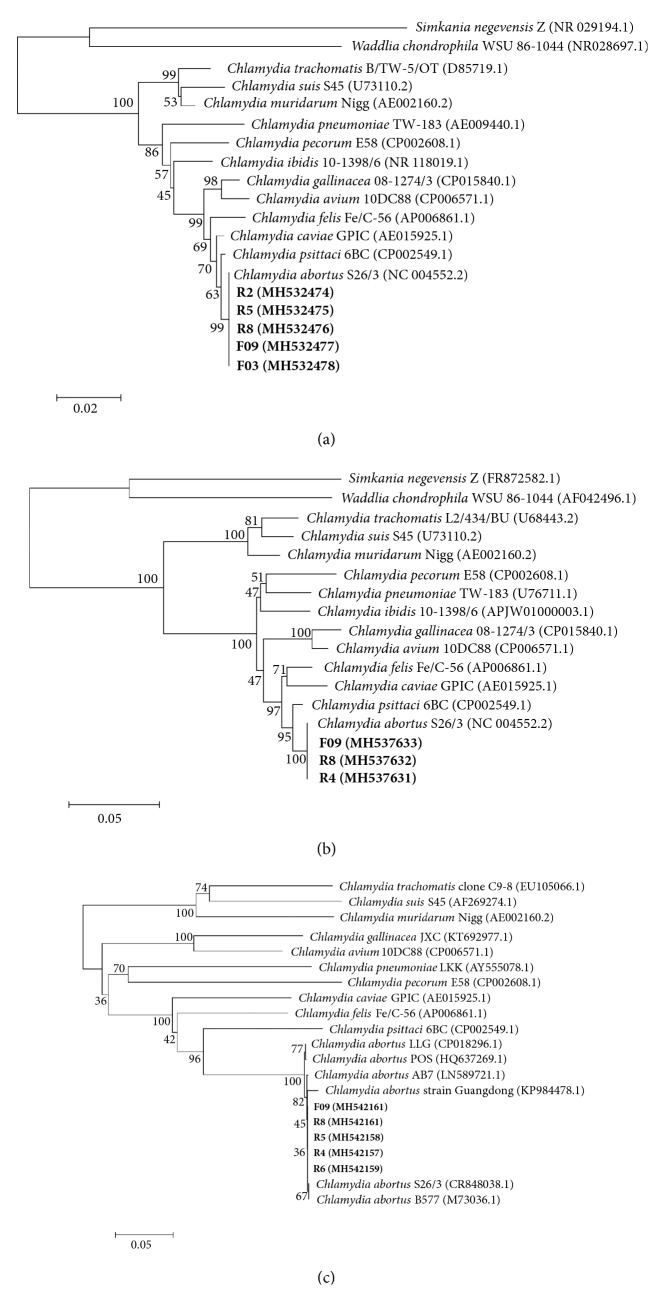
Phylogenetic tree based on 16S rRNA gene fragment (1358 bp) (a), 16S-23S intergenic spacer and full length of 23S rRNA domain I fragment (990 bp) (b), and* ompA* gene fragment (940 bp) (c). Representative sequences of established* Chlamydiaceae* species as well as the sequences obtained from this study (Genbank accession number was shown in bold) were included.* S*.* negevensis *strain Z was used as an outgroup. Based on these alignments, phylogenetic trees were constructed by the neighbor-joining method using the Maximum Composite Likelihood model with MEGA 5.05.

To further confirm the presence of* Chlamydia *spp., amplification and sequencing of 16S rRNA, IGS-23S rRNA, and* ompA* gene fragments were carried out on high positive samples. The 16S rRNA gene amplicons were successfully obtained from 2 samples originating from foxes (MH532477, MH532478) and 3 samples originating from raccoon dogs (MH532474-MH532476), IGS-23S rRNA from 1 fox (MH537633) and 2 samples from raccoon dogs (MH537631, MH537632), and* ompA* from 1 fox (MH542161) and 4 samples from raccoon dogs (MH542157-MH542160), while the process of obtaining amplicons from mink samples failed. Dendrograms were constructed on the basis of* ompA*, 16S rRNA, and IGS-23S rRNA gene fragments aligning with* Chlamydia *species reference sequences available in GenBank and altogether showed a similar topology. All sequences obtained in this study were assigned to* C. abortus* within the* Chlamydiaceae* family (Figure 1). In addition, phylogenetic analysis showed* ompA* sequences were clustered with the strains* C. abortus* S26/3 and B577 hosted by ruminants as the closest relative.

Chlamydiosis is common in livestock, poultry, companion, and wild animals [19, 20]. In the available literature, there is a lack of reports about chlamydiosis cases in fur animals. In this study, evidence for the existence of* C. abortus* in farmed fur animals was shown for the first time. It seemed that the gastrointestinal tract provided a major endemic habitat for* C. abortus* in farmed foxes and raccoon dogs, although the genital tract was generally considered as the colonized site for this pathogen in female animals. Another* Chlamydia* species found in this study as a coexisting pathogen in a few rectal swabs in foxes was* C. pecorum*, pathogen of the koala, and commonly found in the gastrointestinal tract of cattle [21, 22]. These results suggested that* C. abortus *was the dominant* Chlamydia* species carried by tested animals. However, due to the limited number of samples investigated in this study, more evidence is required to reveal the relationship between* C. abortus *infection and reproductive disorder in foxes and raccoon dogs. In addition, it is not clear whether fur animals are the natural hosts of* Chlamydia* spp. such as* C. abortus* or if infection occurs via other vectors. It is worth mentioning that the feed for fur animals in China always contains fish, chicken, and occasionally pig products. As has been reported, the prevalence of* Chlamydia* spp. in chickens and pigs is quite common in China [23, 24]. Therefore, feeding should be considered as a possible way for farmed fur animals to become infected with* Chlamydia* spp.

*C. abortus* is well known for its zoonotic nature. Frequent human contact with domestic livestock at work increases the risk of infection. According to data published in 2002, the average number of notified cases of human chlamydiosis originating from* C. abortus* infected animals was approximately 100 per year in Germany [25]. Although the possible transmission of* C. abortus* from farmed fur animals to humans has not been investigated yet, environmental contamination by the faecal shedding of this zoonotic agent may become a potential source of infection for workers exposed to infected animals during their daily activities.

## 4. Conclusions

This is the first report confirming that farmed fur animals, foxes, raccoon dogs, and minks, mainly harbored* C. abortus*, while* C. pecorum* occasionally coexists in foxes. Further studies are needed to fully elucidate the epidemiology and pathogenicity of* C. abortus* in farmed fur animals. Moreover, an evaluation of the potential health risk to fur farm personnel and the creation of a strategy to preserve human safety are required.

## Figures and Tables

**Table 1:** Results summary of farmed fur animals testing.

species of animals	Gender	no. of tested /*Chlamydiaceae* positive samples				no. of *C. abortus* positive/*Chlamydiaceae* positive samples				no. of *C. pecorum* positive/* Chlamydiaceae* positive samples			
		type of sample											
		rectal swabs	blood	vaginal swabs	conjunctive swabs	rectal swab	blood	vaginal swabs	conjunctive swabs	rectal swabs	blood	vaginal swabs	conjunctive swabs
fox	Male	9/9	9/2	—	9/7	9/9	2/2	—	7/7	1/9	0/2	—	0/7
	Female	11/11	11/5	11/9	11/9	11/11	5/5	9/9	9/9	6/11	0/5	0/9	0/9
	Total	20/20	20/7	11/9	20/16	20/20	7/7	9/9	16/16	7/20	0/7	0/9	0/16

raccoon dog	Male	12/12	12/2	—	12/6	12/12	2/2	—	6/6	0/12	0/2	—	0/6
	Female	3/3	3/1	3/1	3/2	3/3	1/1	1/1	2/2	0/3	0/1	0/1	0/2
	Total	15/15	15/3	3/1	15/8	15/15	3/3	1/1	8/8	0/15	0/3	0/1	0/8

mink	Male	16/1	16/0	—	—	1/1	N/A	—	—	0/1	N/A	—	—
	Female	4/0	4/0	4/0	—	N/A	N/A	N/A	—	N/A	N/A	N/A	—
	Total	20/1	20/0	4/0	—	1/1	N/A	N/A	—	0/1	N/A	N/A	—

Note: N/A: not investigated; —: no sample collected.

## Data Availability

The data used to support the findings of this study are available from the corresponding author upon request.

## Supplementary Materials

Supplementary Materials Table S1: summary of real-time PCR results.
