# Supplementary material for: First Report of Chlamydia abortus in Farmed Fur Animals
Source: Biomed Res Int. 2018 Nov 26;2018:4289648. doi: 10.1155/2018/4289648 (PMC6287152; doi:10.1155/2018/4289648)
Supplement: Supplementary Materials — Table S1: summary of real-time PCR results. [file 4289648.f1.docx]

Table S1: Summary of real-time PCR results.

| **species of animal** | **gender** | **animal ID** | **type of sample** | | | |
| --- | --- | --- | --- | --- | --- | --- |
|  |  |  | **rectal swabs**  **(*Chlamydiaceae* / *C. abortus* / *C. pecorum*)** | **blood**  **(*Chlamydiaceae* / *C. abortus*)** | **conjunctive swab**  **(*Chlamydiaceae / C. abortus*)** | **vaginal swab**  **（*Chlamydiaceae* / *C. abortus*）** |
|  |  |  | **copies/μl** | | | |
| **fox** | male | F01 | 83.43 / 54.57 / 123.50 | ND | 52.78 / 37.6541.74 | — |
|  |  | F02 | 81.67 / 31.05 / ND | ND | 46.73 / 38.71 | — |
|  |  | F03 | 99.28 / 37.11 / ND | 80.04 / 48.69 | 87.17 / 77.90 | — |
|  |  | F06 | 68.64 / 52.96 / ND | ND | 61.16 / 53.14 | — |
|  |  | F10 | 86.27 / 60.80 / ND | ND | ND | — |
|  |  | F11 | 86.10 / 34.61 / ND | ND | ND | — |
|  |  | F12 | 91.98 / 42.63 / ND | 88.06 / 86.10 | 34.79 / 20.54 | — |
|  |  | F15 | 70.60 / 72.20 / ND | ND | 49.40 / 37.46 | — |
|  |  | F17 | 79.86 / 46.73 / ND | ND | 83.96 / 78.79 | — |
|  | female | F04 | 84.85 / 55.81 / ND | ND | 64.01 / 53.14 | 66.15 / 25.71 |
|  |  | F05 | 51.18 / 43.88 / 70.85 | 70.42 / 75.59 | 36.40 / 37.01 | ND |
|  |  | F07 | 85.71 / 46.37 / 68.52 | 97.14 / 89.13 | 72.04 / 79.68 | 54.39 / 57.06 |
|  |  | F08 | 88.77 / 52.43 / 70.85 | ND | 62.58 / 59.38 | 67.93 / 30.69 |
|  |  | F09 | 101.24 / 28.91 / 72.47 | ND | ND | 118.52 / 100.35 |
|  |  | F13 | 97.68 / 65.08 / ND | ND | ND | 64.72 / 57.42 |
|  |  | F14 | 78.97 / 38.00 / 85.77 | ND | 67.39 / 49.58 | 81.47 / 87.52 |
|  |  | F16 | 53.50 / 39.78 / 82.71 | ND | 70.60 / 60.80 | 64.36 / 21.79 |
|  |  | F18 | 82.00 / 34.26 / ND | 84.85 / 97.50 | 86.99 / 69.53 | 67.04 / 54.92 |
|  |  | F19 | 63.65 / 41.92 / ND | 34.52 / 83.07 | 78.08 / 51.18 | ND |
|  |  | F20 | 19.29 / 61.16 / ND | 68.10 / 92.16 | 73.09 / 53.68 | 72.56 / 60.80 |

| **species of animal** | **gender** | **animal ID** | **type of sample** | | | |
| --- | --- | --- | --- | --- | --- | --- |
|  |  |  | **rectal swabs**  **(*Chlamydiaceae* / *C. abortus* / *C. pecorum*)** | **blood**  **( *Chlamydiaceae*)** | **conjunctive swab**  **(*Chlamydiaceae / C. abortus*)** | **vaginal swab**  **（*Chlamydiaceae* / *C. abortus*）** |
|  |  |  | **copies/μl** | | | |
| **mink** | male | M01 | ND | ND | — | — |
|  |  | M02 | ND | ND | — | — |
|  |  | M03 | ND | ND | — | — |
|  |  | M04 | 57.95 / 42.10 / ND | ND | — | — |
|  |  | M05 | ND | ND | — | — |
|  |  | M06 | ND | ND | — | — |
|  |  | M07 | ND | ND | — | — |
|  |  | M08 | ND | ND | — | — |
|  |  | M09 | ND | ND | — | — |
|  |  | M10 | ND | ND | — | — |
|  |  | M13 | ND | ND | — | — |
|  |  | M14 | ND | ND | — | — |
|  |  | M15 | ND | ND | — | — |
|  |  | M16 | ND | ND | — | — |
|  |  | M17 | ND | ND | — | — |
|  |  | M19 | ND | ND | — | — |
|  | female | M11 | ND | ND | — | ND |
|  |  | M12 | ND | ND | — | ND |
|  |  | M18 | ND | ND | — | ND |
|  |  | M20 | ND | ND | — | ND |

| **species of animal** | **gender** | **animal ID** | **type of sample** | | | |
| --- | --- | --- | --- | --- | --- | --- |
|  |  |  | **rectal swabs**  **(*Chlamydiaceae* / *C. abortus* / *C. pecorum*)** | **blood**  **( *Chlamydiaceae* / *C. abortus*)** | **conjunctive swab**  **(*Chlamydiaceae / C. abortus*)** | **vaginal swab**  **(*Chlamydiaceae* / *C. abortus*)** |
|  |  |  | **copies/μl** | | | |
| **raccoon dog** | male | R01 | 86.81 / 67.93 / ND | ND | 68.28 / 59.55 | — |
|  |  | R02 | 206.88 / 176.24 / ND | ND | 48.51 / 34.97 | — |
|  |  | R04 | 175.71/ 158.78 / ND | ND | 72.56 / 78.97 | — |
|  |  | R06 | 167.69/ 151.66 / ND | ND | ND | — |
|  |  | R07 | 135.80 / 94.29 / ND | ND | ND | — |
|  |  | R08 | 122.62 / 123.68 / ND | ND | ND | — |
|  |  | R09 | 145.60 / 123.33 / ND | ND | ND | — |
|  |  | R10 | 138.12 / 120.12 / ND | ND | ND | — |
|  |  | R11 | 143.28 / 120.66 / ND | 57.95 / 38.71 | 82.54 / 71.13 | — |
|  |  | R12 | 94.83 / 91.09 / ND | ND | ND | — |
|  |  | R13 | 124.76 / 124.58 / ND | ND | 71.31 / 51.89 | — |
|  |  | R14 | 141.50 / 106.41 / ND | 68.10 / 73.81 | 45.84 / 56.53 | — |
|  | female | R03 | 190.49 / 176.42 / ND | ND | 62.58 / 57.60 | ND |
|  |  | R05 | 163.06 / 148.81 / ND | ND | ND | ND |
|  |  | R15 | 104.09 / 89.48 / ND | 90.37 / 69.00 | 49.58 / 44.59 | 36.02 / 36.52 |
